# Supplementary figures and images for: The association of POSTN with postoperative recurrence risk in early-stage lung adenocarcinoma: From gene networks to cellular functions
Source: PLoS One. 2025 Sep 24;20(9):e0331590. doi: 10.1371/journal.pone.0331590 (PMC12459812; doi:10.1371/journal.pone.0331590)

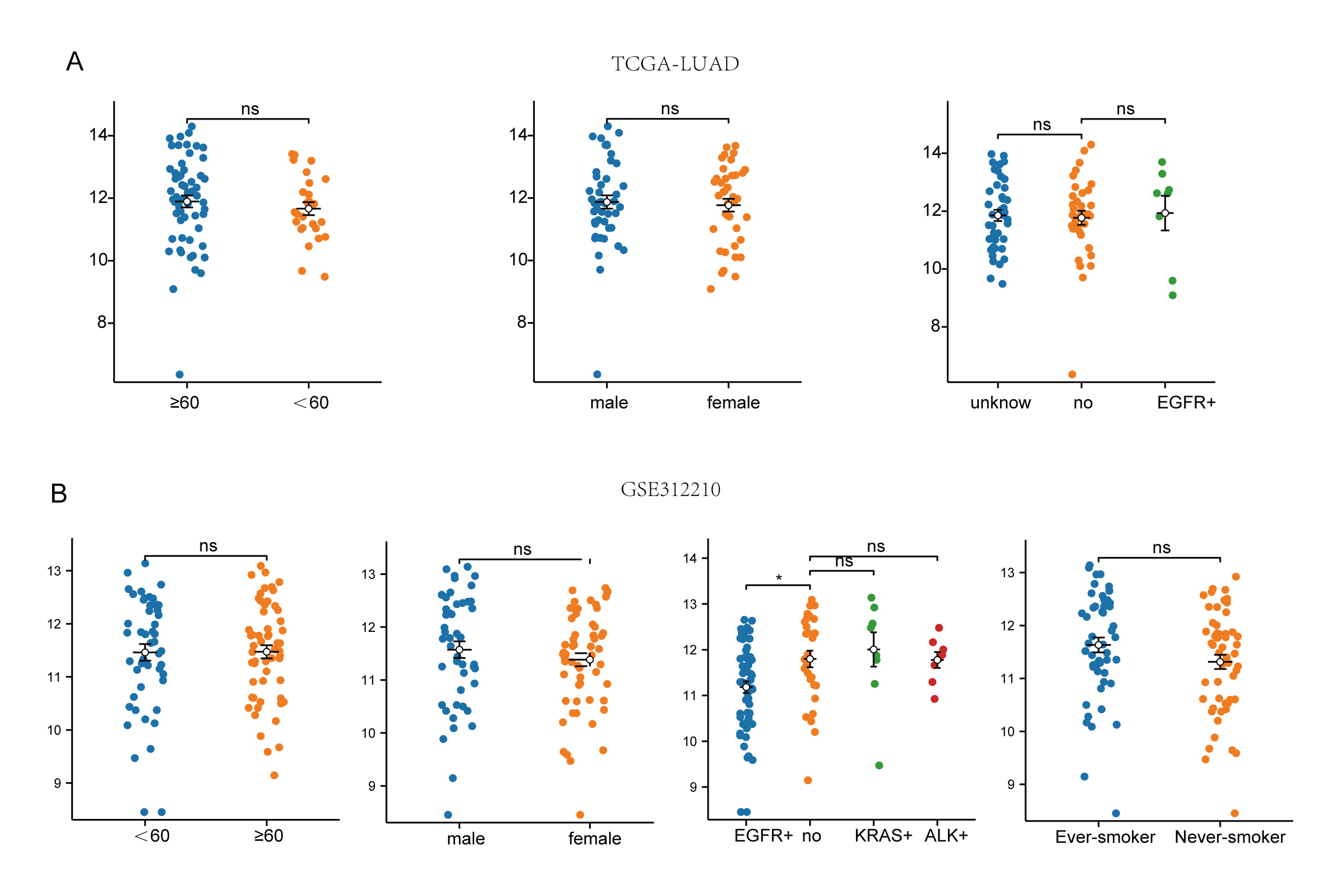

Supplement: S1 Fig — (A) The distribution of POSTN in different variable populations in the TCGA database. (B) The distribution of POSTN in different variable populations in the GEO dataset. (TIF) [file pone.0331590.s001.tif]
