# Supplementary material for: The association of POSTN with postoperative recurrence risk in early-stage lung adenocarcinoma: From gene networks to cellular functions
Source: PLoS One. 2025 Sep 24;20(9):e0331590. doi: 10.1371/journal.pone.0331590 (PMC12459812; doi:10.1371/journal.pone.0331590)
Supplement: S1 Table — (DOCX) [file pone.0331590.s002.docx]

| **S1 Table Abbreviations and Full Names of Words** | |
| --- | --- |
| Abbreviation | Full Name |
| AGTR1 | angiotensin II receptor 1 |
| AUC | Area Under the Curve |
| CAF | Cancer-Associated Fibroblast |
| CI | Confidence Interval |
| CRSwNP | Chronic Rhinosinusitis with Nasal Polyps |
| DMEM | Dulbecco’s Modified Eagle Medium |
| ECM | Extracellular Matrix |
| EMT | Epithelial-Mesenchymal Transition |
| ESCC | Esophageal Squamous Cell Carcinoma |
| FBS | Fetal Bovine Serum |
| GEO | Gene Expression Omnibus |
| GO | Gene Ontology |
| GSEA | Gene Set Enrichment Analysis |
| KM | Kaplan-Meier |
| LUAD | Lung Adenocarcinoma |
| N | Number |
| NSCLC | Non-Small Cell Lung Cancer |
| OR | Odds Ratio |
| OE_NC | Overexpression Negative Control |
| OE_POSTN | Overexpression of POSTN |
| OS | Overall Survival |
| PBS | Phosphate-Buffered Saline |
| PFI | Progression-Free Interval |
| POSTN | Periostin |
| ROC | Receiver Operating Characteristic |
| mCRPC | metastatic Castration-Resistant Prostate Cancer |
| M±SD | Mean ± Standard Deviation |
| siPOSTN | POSTN-targeting siRNA |
| STAS | spreading through air space |
| TCGA | The Cancer Genome Atlas |
| TOP2A | Topoisomerase IIA |
| WGCNA | Weighted Gene Co-expression Network Analysis |
