# Supplementary material for: The association of POSTN with postoperative recurrence risk in early-stage lung adenocarcinoma: From gene networks to cellular functions
Source: PLoS One. 2025 Sep 24;20(9):e0331590. doi: 10.1371/journal.pone.0331590 (PMC12459812; doi:10.1371/journal.pone.0331590)
Supplement: S2 Table — (DOCX) [file pone.0331590.s003.docx]

| **S2 Table Data Inclusion and Analysis Purpose** | | | | | |
| --- | --- | --- | --- | --- | --- |
| Analysis module | Original dataset | Original sample size | The actual sample size included | Definition of clinical endpoints | Main exclusion/stratification criteria |
| WGCNA + single-gene verification | TCGA-LUAD | 426 | 84 | PFI <1 yr vs ≥5 yr | Pathological stage I-II, radical resection, complete follow-up |
| External validation 1 | GSE31210 | 226 | 105 | PFI <1 yr vs ≥5 yr | Complete follow-up |
| External validation 2 (invasiveness) | GSE166722 | 53 | 53 | High invasiveness vs low invasiveness | Pathological stage I-II |
| Analysis of clinical efficacy | TCGA + GSE31210 | 310 | 189（TCGA=84，GSE31210=105） | PFI <1 yr vs ≥5 yr |  |
