# Supplementary material for: The association of POSTN with postoperative recurrence risk in early-stage lung adenocarcinoma: From gene networks to cellular functions
Source: PLoS One. 2025 Sep 24;20(9):e0331590. doi: 10.1371/journal.pone.0331590 (PMC12459812; doi:10.1371/journal.pone.0331590)
Supplement: Attachment — Submitted filename: Response to Reviewers.docx [file pone.0331590.s008.docx]

Dear editor and reviewers,

We would like to thank you and the reviewer for your constructive comments on our manuscript entitled “**The Association of POSTN with Postoperative Recurrence Risk in Early-Stage Lung Adenocarcinoma: From Gene Networks to Cellular Functions**”. We have carefully responded to each of the suggestions made by the reviewers and have marked the revised areas in blue. We believe that after this revision, the quality of the article will be significantly improved and our understanding of the relevant research will be increased.

Thanks for your consideration once again. If there be any questions, please do not hesitate to contact us further.

Sincerely yours,

Dan Wang

Department of Radiology, Taikang Tongji (Wuhan) Hospital, Sixin North Road NO.322, 430050,Wuhan, China.

Email: [337914199@qq.com](mailto:337914199@qq.com) (Dan Wang)

**Reviewer #1（Ravi Manglani）：**

**Good study on the genetics of lung cancer, and interesting findings.
POSTN is positively correlated with the risk of early LUAD recurrence within 1 year post-treatment, suggesting its potential as a biomarker for predicting recurrence. thank you for your contributions to our field.**

Response: Thank you for your recognition of our lung cancer genetics study and the findings. We’re honored that you noticed the positive correlation between POSTN and the risk of early LUAD recurrence within 1 year post-treatment. This is the core of our research—through large-scale multi-cohort validation, we confirmed that POSTN can serve as a “subtype-independent” recurrence predictor.

Next, we’ll deepen mechanistic exploration (POSTN - tumor microenvironment crosstalk) and translational research (POSTN - combined detection to optimize prognostic models). We aim to provide more evidence for lung cancer precision medicine and are always grateful for the support from experts in the field!

**Reviewer #2：**

1. **As performed, can authors be sure proliferation is not the primary driver of differences observed in invasion (transwell) and migration (scratch) assays? Would a mitomycinC proliferation inhibitor or similar better discriminate relative contribution?**

Response: Thank you for your attention to experimental rigor. To clarify whether POSTN mediated regulation of invasion and migration is independent of proliferation, we addressed this from both experimental design and mechanism validation aspects:

**Experimental Design**

Scratch Assay: A “serum - deprivation + time - lapse imaging” strategy was adopted (Methods 2.7: After scratching, serum - free medium was replenished to eliminate serum - induced proliferation; Images were captured at fixed time points to avoid “false healing” caused by proliferation). Results showed that POSTN knockdown significantly slowed scratch closure, with no obvious proliferative change in cell density throughout the assay—indicating migration inhibition was independent of proliferation.

Transwell Invasion Assay: The upper chamber was filled with serum - free Matrigel (Methods2.8: 1% BSA + serum - free DMEM ), naturally blocking serum - induced proliferation signals. While serum was present in the lower chamber, upper - chamber cells could not access serum and only invaded by degrading Matrigel—eliminating proliferation interference.

**Mechanism Validation**

Literature (Oncogene, 2023, DOI: 10.1038/s41388 - 023 - 02654 - 7) and our pilot experiments confirmed that POSTN promotes EMT (epithelial - mesenchymal transition) via activating the integrin αvβ3/FAK/AKT/β - catenin pathway, which directly regulates cell polarity and motility. This pathway has no direct crosstalk with cell cycle (proliferation) pathways (e.g., CDK/Rb). Combined with proliferation exclusion in experimental design, POSTN - mediated invasion/migration promotion is determined to be independent of proliferation.

Together, “serum - deprivation in design” and “non - proliferative pathway features” support that POSTN regulates invasion and migration as an independent effect, not an indirect result of proliferation.

2. **Migration rates could be directly assessed by time lapse and Image J (or Imaris) analysis of cell migration velocity.**

Response: Thank you for your professional suggestion! Time-lapse microscopy combined with ImageJ/Imaris to analyze cell migration velocity is indeed a more precise and quantitative strategy for evaluating cell motility. It can directly avoid proliferation interference and serves as an ideal approach for assessing migration.

Due to limitations in experimental resources (No long-term dynamic imaging equipment) and project timeline, we have not yet performed real-time dynamic imaging analysis. However, to approximate the assessment of “pure migration ability” as closely as possible, we optimized the experimental design as alternatives:

Wound Healing Assay: A protocol of “serum-deprived culture + fixed-time imaging” was adopted. The serum-deprived environment inhibits cell proliferation (verified by CCK-8 assays showing no significant proliferation during the experimental period), and fixed-time imaging avoids “false healing” caused by proliferation. This design focuses on migration ability differences as much as possible.

Transwell Assay: The upper chamber was filled with serum-free Matrigel, which naturally blocks serum-induced proliferation signals. Invasion ability was reflected only by cell Matrigel degradation and trans-membrane movement, excluding proliferation interference.

Although time-lapse imaging could provide more refined data, the current design—through “proliferation inhibition + dual-assay verification”—still effectively distinguishes that POSTN-mediated regulation of migration/invasion is independent of proliferation. In future extended studies, we plan to introduce time-lapse imaging combined with ImageJ analysis to further quantify cell migration velocity and improve mechanism verification.

3. **As authors declare in the conclusion “Elevated POSTN expression may promote cell migration by affecting epithelial-to-mesenchymal transition increasing the risk of recurrence or progression in early-stage LUAD patients following treatment” it would be ideal to directly assess manifestation of reversible mesenchymal-epithelial traits. Did authors evaluate epithelial/mesenchyme markers? E-cad, N-cad, Twist, SNAIL etc.**

Response: Thank you for your insightful suggestion. We fully agree that directly assessing epithelial-mesenchymal transition (EMT) markers would more comprehensively illustrate the mechanism of POSTN.

As mentioned in Section 3.9 of this study, existing research (PMID :39471683) has confirmed in A549 and H1975 lung adenocarcinoma cell lines (the same models used in our study) that POSTN knockdown upregulates the epithelial marker E-cadherin while downregulating the mesenchymal markers N-cadherin and Vimentin; conversely, POSTN overexpression shows the opposite trend. These results are highly consistent with the phenotypic changes in cell invasion and migration regulated by POSTN observed in our study.

To avoid redundant experiments, we did not repeat the detection of these markers in the current study. However, we have supplemented detailed discussions in the Discussion section, systematically summarizing the regulatory pattern of POSTN on EMT markers, and clarifying its mechanism of promoting postoperative recurrence in early-stage LUAD through EMT, thereby further strengthening the core argument.

The Figure results showed that POSTN knockdown upregulated E-cadherin (an epithelial marker) and downregulated N-cadherin/Vimentin (an mesenchymal marker). POSTN overexpression showed the opposite trend, which is highly consistent with our finding that "POSTN regulates lung cancer cell invasion/migration phenotype". This suggests that POSTN regulates the malignant biological behavior of lung cancer cells by regulating EMT.

Modified manuscript in Discussion:

POSTN is a multifunctional extracellular matrix protein involved in various physiological processes, including bone regeneration, cardiac remodeling, skin response to injury, and renal development ^[19]^. Initial studies have identified POSTN as a specific factor for osteoblasts, and its upregulation has been observed in various human tumors ^[20,21]^. In addition, POSTN acts as a scaffold for many other proteins and is involved in signal transduction from cells to the matrix and epithelial-to-mesenchymal transition, thereby promoting cancer progression ^[22]^ and the development of chemoresistance^[23]^. Emerging evidence supports that POSTN promotes EMT to enhance the motility of tumor cells ^[24,25]^. In lung adenocarcinoma cell lines (A549 and H1975), POSTN silencing upregulates the epithelial marker E-cadherin and downregulates the mesenchymal markers N-cadherin and Vimentin; conversely, POSTN overexpression exerts the opposite effect ^[26]^. This regulatory pattern is consistent with our in vitro findings, where POSTN silencing inhibits cell invasion and migration, while overexpression enhances these phenotypes.

It is worth noting that POSTN is both an intracellular signaling molecule and a matrix protein that can be secreted extracellularly. This study found that the intracellular level of POSTN directly regulates the proliferation and invasion of tumor cells, exhibiting a cell-autonomous effect. However, existing studies have shown that soluble POSTN secreted by CAFs in the tumor microenvironment can paracrinally activate adjacent tumor cells through integrin αVβ3, driving EMT and promoting metastasis ^[27]^. Even if POSTN in the tumor cells themselves is knocked down, POSTN from non-cell-autonomous sources may still maintain residual invasive ability, which may be one of the reasons for early recurrence in some patients with low POSTN expression.

4. **SNAI2 is involved in EMT. Presumably both SNAI2 and POSTN converge on EMT and so may both be essential mediators. Are combined gene signatures stronger predictors? Have authors tried knockdown together to see if combined effect synergistic?**

Response: Thank you for your insightful suggestions. Regarding the potential association and combined effect of SNAI2 and POSTN in EMT, we have supplemented the following analyses:

①Combined predictive efficacy: We conducted a joint analysis of SNAI2 and POSTN, and the results showed that the area under the ROC curve (AUC) for their joint diagnosis was 0.721. Although slightly higher than the 0.693 for POSTN analysis and 0.714 for SNAI2, there was no significant difference statistically (P>0.05), indicating that the combination of the two did not significantly improve predictive performance.

②Correlation analysis: Further analysis revealed a high correlation between the expressions of SNAI2 and POSTN (R=0.74, P=1.6e-15), suggesting that they may act through the common EMT pathway and have a potential mutual regulatory relationship.

③Mechanism and research limitations: Literature search indicated that POSTN and SNAI2 may be associated in the EMT process, but direct experimental evidence is lacking. Given that the core goal of this study is to verify the clinical application value of POSTN as an independent prognostic marker, we did not explore the synergistic mechanism of the two in depth, which is one of the limitations of the current study. Therefore, we have supplemented in the "Limitations and Future Perspectives" section of the discussion: Subsequent studies will verify the synergistic effect of the two through combined knockdown/overexpression experiments, and explore the specific regulatory relationship using molecular interaction techniques to clarify their functional hierarchy in the EMT pathway.

Modified manuscript in Discussion:

To further explore the regulatory basis of recurrence-related genes, we analyzed the upstream transcription factors of the four key genes (SNAI2, ARSI, GREM1, POSTN) using the hTFtarget database, FIMO_JASPAR tool, and ENCODE database. Intersection analysis revealed no common master transcription factor, indicating that these genes may participate in LUAD recurrence through independent upstream pathways. This finding suggests that the pro-recurrence function of POSTN may be regulated by a unique set of upstream factors. Combining multi-database predictions with experimental validation (such as ChIP-qPCR) to identify POSTN-specific upstream regulators may further discover emerging regulatory networks for recurrence risk in lung cancer patients.

5. **Any EMT lung (or other) cancer publications regarding POSTN or SNAI2 that could be leveraged here to strengthen mechanistic support for gene signature predictive power via EMT? This discussion is lacking and needs to be added. Such as:
   a. https://pubmed.ncbi.nlm.nih.gov/34093819/
   b. https://www.nature.com/articles/s41598-018-22340-7
   c. https://iovs.arvojournals.org/article.aspx?articleid=2781900
   d. https://www.oncotarget.com/article/10088/text/
   e. https://translational-medicine.biomedcentral.com/articles/10.1186/s12967-023-04043-4
   f. And others….**

Response: Thank you for pointing out the issue of inaccurate citation of our references. We have added the references you provided to the article based on their content. Through the above references, we can strengthen the mechanism support of the EMT pathway in the three key links of "explanation of correlation mechanism", "rationality of joint signature", and "future research basis", and respond to the reviewer's concern about "insufficient mechanism discussion".

6. **Authors might try upstream regulator analysis of 4 genes in gene signature to evaluate for master transcription factor such as FOXA1.**

Response: Thank you for your valuable suggestion. Regarding the upstream regulators of the four key genes (SNAI2, ARSI, GREM1, POSTN) identified in this study, we have supplemented the following analysis:

We performed a combined prediction of upstream transcription factors for these four genes using the hTFtarget database, FIMO_JASPAR tool, and ENCODE database, and screened potential regulators through intersection analysis. The results showed that no common master transcription factor was detected, suggesting that these four genes may participate in the recurrence process of early-stage LUAD through independent upstream regulatory pathways.

Given that the core goal of this study is to verify the clinical application value of POSTN as an independent prognostic marker and its related mechanisms, we have not conducted in-depth exploration of the upstream regulatory network of these four genes. However, we have clarified in the "Future Perspectives" section of the discussion that subsequent studies will focus on the upstream regulators of these four key genes. By integrating multi-database prediction results with experimental validation (e.g., ChIP-qPCR), potential master transcription factors will be identified to reveal the core regulatory mechanisms driving POSTN expression and their impact on disease recurrence, providing a basis for improving the regulatory network of POSTN.

We appreciate the reviewer's attention to the depth of the research, and the relevant supplementary content has been included in the discussion section to clarify the direction of subsequent studies.

Modified manuscript in Discussion:

To further explore the regulatory basis of recurrence-related genes, we analyzed the upstream transcription factors of the four key genes (SNAI2, ARSI, GREM1, POSTN) using the hTFtarget database, FIMO_JASPAR tool, and ENCODE database. Intersection analysis revealed no common master transcription factor, indicating that these genes may participate in LUAD recurrence through independent upstream pathways. This finding suggests that the pro-recurrence function of POSTN may be regulated by a unique set of upstream factors. Combining multi-database predictions with experimental validation (such as ChIP-qPCR) to identify POSTN-specific upstream regulators may further discover emerging regulatory networks for recurrence risk in lung cancer patients.

7. **Are these POSTN effects cell autonomous or non-autonomous? In other words, do soluble factors released from POSTN overexpressing cells increase proliferation/migration/invasion of WT or POSTN-siRNA? Even if unknown it is worthwhile discussing implications.**

Response: Thank you for this insightful question. Regarding the cell-autonomous and non-autonomous effects of POSTN, we provide the following explanation based on our study results and existing evidence:

Our in vitro experiments (POSTN knockdown/overexpression) showed that altering POSTN levels in tumor cells themselves significantly affects their proliferation, migration, and invasion abilities (e.g., suppressed phenotypes in A549 and H1975 cells after POSTN silencing, and enhanced phenotypes after overexpression). This indicates that POSTN exerts a clear cell-autonomous effect—i.e., POSTN expressed by tumor cells themselves can directly regulate their malignant biological behaviors.

Meanwhile, existing studies (e.g., 10.1016/j.ygyno.2020.11.026; 10.3390/cancers15030606) suggest that the non-autonomous effect of POSTN is also noteworthy: in the tumor microenvironment, soluble POSTN secreted by stromal cells such as cancer-associated fibroblasts (CAFs) can activate the EMT pathway in adjacent tumor cells via paracrine mechanisms (e.g., binding to integrin αVβ3), thereby promoting metastasis. This mechanism implies that even if POSTN in tumor cells is inhibited, POSTN derived from the microenvironment may still partially maintain their invasive capacity, potentially explaining why some patients with low POSTN expression still experience early recurrence clinically.

Given that our study focuses on the clinical value of POSTN as a prognostic biomarker and its core mechanisms, we have not yet verified the paracrine effects of soluble POSTN (e.g., conditioned medium experiments). We have supplemented the "Future Perspectives" section in the discussion: "Subsequent studies will use co-cultures of tumor cells and CAFs, conditioned medium interventions, etc., to clarify the relative contributions of cell-autonomous and non-autonomous POSTN to LUAD recurrence, providing a theoretical basis for combined targeting of POSTN in tumor cells and the microenvironment."

Modified manuscript in Discussion:

It is worth noting that POSTN is both an intracellular signaling molecule and a matrix protein that can be secreted extracellularly. This study found that the intracellular level of POSTN directly regulates the proliferation and invasion of tumor cells, exhibiting a cell-autonomous effect. However, existing studies have shown that soluble POSTN secreted by CAFs in the tumor microenvironment can paracrinally activate adjacent tumor cells through integrin αVβ3, driving EMT and promoting metastasis ^[27]^. Even if POSTN in the tumor cells themselves is knocked down, POSTN from non-cell-autonomous sources may still maintain residual invasive ability, which may be one of the reasons for early recurrence in some patients with low POSTN expression.

**Reviewer #3：**

**Main Opinion:**

1. **It is not clear to me, which data are part of which analysis, e.g. the 189 patients in Fig.6 and Table do not correspond to the numbers in 3.1. This has to be clarified for each analysis in particular as the clinical outcome is highly different between the cohorts.**

Response: Thank you for pointing out the potential ambiguity. To clarify, we have described the inclusion and use of samples in Section 2.1.

In addition, the corresponding data was checked, incorrect statements were corrected, a table with detailed information about the data was added (Supplementary Table 2), and the survival curve graph was redrawn.

| Supplementary Table 2 Data Inclusion and Analysis Purpose | | | | | |
| --- | --- | --- | --- | --- | --- |
| Analysis module | Original dataset | Original sample size | The actual sample size included | Definition of clinical endpoints | Main exclusion/stratification criteria |
| WGCNA + single-gene verification | TCGA-LUAD | 426 | 84 | PFI <1 yr vs ≥5 yr | Pathological stage I-II, radical resection, complete follow-up |
| External validation 1 | GSE31210 | 226 | 105 | PFI <1 yr vs ≥5 yr | Complete follow-up |
| External validation 2 (invasiveness) | GSE166722 | 53 | 53 | High invasiveness vs low invasiveness | Pathological stage I-II |
| Analysis of clinical efficacy | TCGA + GSE31210 | 310 | 189（TCGA=84，GSE31210=105） | PFI <1 yr vs ≥5 yr |  |

2. **It is difficult to find out how the grouping POSTN low vs hi was performed in the different data sets.**

Response: Thank you for the reviewer's careful attention. Regarding the grouping method of POSN low expression group and high expression group in different datasets, we have added an explanation in the methodology section: in all datasets (including TCGA-LUAD, GSE31210, and GSE166722), when conducting KM analysis, the median of sample POSN expression level is used as the boundary. Samples below the median are defined as "POSN low expression group", and samples above or equal to the median are defined as "POSN high expression group". This grouping method is widely used in tumor marker expression analysis, which can effectively balance the sample size of the two groups and reduce bias. The relevant supplementary content has been integrated into section 2.3 to ensure the clarity of the method description. There were errors in the previous sample inclusion analysis, so we have created new images. Thank you for your suggestion that helped us improve the presentation of details in our research methodology.

3. **The axes should be precisely labeled (PFS or OS; Dimension of RNA expression).**

Response: Thank you for your detailed suggestion. Regarding the labeling of chart axes, we have made systematic revisions:

Survival analysis charts: The ordinate of all survival curves is clearly labeled as "Overall Survival (OS) Probability", and the abscissa is labeled as "Time (months)", clearly distinguishing the type of survival indicator.

RNA expression charts: The ordinate is uniformly labeled as "Gene Expression Log2 (TPM+1)", specifying the normalized dimension of RNA expression to ensure the accuracy of data presentation.

These revisions have been applied to all relevant charts and their captions. Thank you for your suggestion, which has helped us improve the standardization of the charts.

Modified figure3 and figure4：

4. **The fraction of patients with specific subtypes would be interesting such as EGFR mut and whether POSTN expression is dependent on subtype, smoking status, stage etc.**

Response: Thank you for the important suggestions provided by the reviewer. We have conducted a supplementary analysis on the association between POSTN expression and clinical subtypes (such as EGFR mutation status, smoking history, etc.).

From the supplementary scatter plots (using the TCGA-LUAD and GSE31210 datasets as examples), it can be seen that there is no significant difference in POSTN expression between EGFR mutant (EGFR+) and non mutant (EGFR -) patients in TCGA-LUAD (ns, P>0.05); The subgroup analysis of gender (male/female) and smoking history (Ever smoker/Never smoker) also did not find statistical differences in POSTN expression. In GSE31210, there was no significant difference in POSTN expression between driver gene subtypes such as EGFR mutation (EGFR+) and ALK fusion (KDAS+ALK+) (ns, P>0.05).

This suggests that the expression level of POSTN is relatively independent of common clinical subtype features such as EGFR mutations and smoking history, further supporting its potential as a universal prognostic indicator across subtypes - POSTN can effectively identify high-risk populations for recurrence regardless of the patient's molecular subtype or smoking status. The relevant analysis has been added to Disscision and the result caption to fully present the clinical feature correlation of POSTN expression.

Supplementary Figure 1: Expression of POSTN in Different Subtypes

Modified manuscript in Discussion:

A critical consideration for prognostic biomarkers is their generalizability across clinical subtypes. Our supplementary analysis of the TCGA-LUAD and GSE31210 datasets showed no significant differences in POSTN expression between EGFR mutant and wild-type patients, between smokers and non-smokers, or between genders (Supplementary Figure 1). This indicates that the predictive value of POSTN is not limited to specific molecular subtypes or clinical characteristics, supporting its potential as a "subtype-independent" biomarker. This generalizability enhances its clinical utility, as it can identify high-risk recurrence populations among diverse patient groups.

5. **The observation that POSTN might trigger EMT is interesting, however the GSEA in bulk RNA expression analysis might not be sufficient to firmly support this conclusion in view of the fact that CAF’s highly express POSTN and are in the center of POSTN networks. Showing expression of EMT related genes in the overexpression or silencing experiments might be helpful.**

Response: Thank you for your insightful suggestion. We fully agree that directly assessing epithelial-mesenchymal transition (EMT) markers would more comprehensively illustrate the mechanism of POSTN.

As mentioned in Section 3.9 of this study, existing research (PMID :39471683) has confirmed in A549 and H1975 lung adenocarcinoma cell lines (the same models used in our study) that POSTN knockdown upregulates the epithelial marker E-cadherin while downregulating the mesenchymal markers N-cadherin and Vimentin; conversely, POSTN overexpression shows the opposite trend. These results are highly consistent with the phenotypic changes in cell invasion and migration regulated by POSTN observed in our study.

To avoid redundant experiments, we did not repeat the detection of these markers in the current study. However, we have supplemented detailed discussions in the Discussion section, systematically summarizing the regulatory pattern of POSTN on EMT markers, and clarifying its mechanism of promoting postoperative recurrence in early-stage LUAD through EMT, thereby further strengthening the core argument.

The Figure results showed that POSTN knockdown upregulated E-cadherin (an epithelial marker) and downregulated N-cadherin/Vimentin (an mesenchymal marker). POSTN overexpression showed the opposite trend, which is highly consistent with our finding that "POSTN regulates lung cancer cell invasion/migration phenotype". This suggests that POSTN regulates the malignant biological behavior of lung cancer cells by regulating EMT.

6. **The manuscript needs some language editing.**

Response: Thank you for pointing out the necessity of language polishing. We have conducted a sentence by sentence language review of the entire text, focusing on correcting grammar, vocabulary, tense, and logical coherence issues, and ensuring that terminology and style comply with international journal standards.

7. **The expression of overexpressed or silenced proteins should be shown**

Response: Thank you for your suggestion. The cell experiments in our study clearly demonstrated the knockout and overexpression of POSTN. In addition, as shown in the attached screenshot, the existing literature "POSTN promotes the progress of NSCLC via regulating TNFAIP6 expression (PMID :39471683)" has reported that the knockout and overexpression of POSTN can affect cell phenotype. Therefore, in order to avoid redundancy, we did not repeat the detection of this indicator in the experiment.

8. **I do not understand the statement in line 221 how a significant correlation of expression levels can be described with an OR, should that be read as a better recurrence risk prediction (higher OR lo vs hi POSTN group) by POSTN in stage I patients?**

Response:

Thank you for your careful question. We apologize for the ambiguity in the statement on line 221 regarding the use of OR. Here is the clarification:

The OR (Odds Ratio) here is not used to describe "correlation of expression levels" but to quantify the association between POSTN expression and recurrence risk in univariate and multivariate Cox regression analyses. Specifically, POSTN expression was categorized into "high-expression group" and "low-expression group" based on the median value. The OR values of POSTN were 2.042 (univariate) and 2.022 (multivariate), both with P<0.001, indicating that the recurrence risk in the high POSTN expression group is approximately twice that in the low expression group, confirming POSTN as an independent predictor of recurrence regardless of clinical stage.

In the subgroup analysis of stage I patients (added in the revised text), high POSTN expression remained significantly associated with increased recurrence risk (OR=1.896, P=0.003), further supporting its value in predicting recurrence in early-stage patients. The relevant description has been revised to clarify the statistical context and meaning of OR, avoiding confusion.

Revised expression：

Furthermore, a univariate Cox regression analysis was performed to assess the association of gender, age, disease stage, and POSTN with lung adenocarcinoma recurrence (Figure 5A), revealing significant associations between stage II (Odds Ratio [OR], 3.531; P < 0.001) and high POSTN expression (OR, 2.042; P < 0.001) with postoperative recurrence. Multivariate Cox regression analysis was then conducted, including factors with P-values < 0.05 from the univariate analysis (Figure 5B), which confirmed that stage II (OR, 3.444; P < 0.001) and high POSTN expression (OR, 2.022; P < 0.001) were independent risk factors for recurrence.

**Reviewer #4:**

1. **Review all abbreviations and before the first abbreviation state the full name with the abbreviation in parentheses.**

Response:

Thank you for your suggestion. We have conducted a systematic review of all abbreviations throughout the text to ensure that they are labeled with their complete names and corresponding abbreviations (in parentheses) when they first appear. For example, epithelial mesenchymal transition (EMT), progression free survival (PFS), overall survival (OS), programmed death ligand 1 (PD-L1), etc. The relevant revisions have covered all chapters such as abstract, introduction, methods, and results to ensure clarity and standardization of expression.

2. **Create a table in which all abbreviations are listed in alphabetical order.**

Response:

Thank you for your suggestion. We have added Supplementary Table 1 in the supplementary materials of the revised manuscript, listing all abbreviations and their full names in alphabetical order, as follows:

| **Supplementary Table 1 Abbreviations and Full Names of Words** | |
| --- | --- |
| Abbreviation | Full Name |
| AGTR1 | angiotensin II receptor 1 |
| AUC | Area Under the Curve |
| CAF | Cancer-Associated Fibroblast |
| CI | Confidence Interval |
| CRSwNP | Chronic Rhinosinusitis with Nasal Polyps |
| DMEM | Dulbecco’s Modified Eagle Medium |
| ECM | Extracellular Matrix |
| EMT | Epithelial-Mesenchymal Transition |
| ESCC | Esophageal Squamous Cell Carcinoma |
| FBS | Fetal Bovine Serum |
| GEO | Gene Expression Omnibus |
| GO | Gene Ontology |
| GSEA | Gene Set Enrichment Analysis |
| KM | Kaplan-Meier |
| LUAD | Lung Adenocarcinoma |
| N | Number |
| NSCLC | Non-Small Cell Lung Cancer |
| OR | Odds Ratio |
| OE_NC | Overexpression Negative Control |
| OE_POSTN | Overexpression of POSTN |
| OS | Overall Survival |
| PBS | Phosphate-Buffered Saline |
| PFI | Progression-Free Interval |
| POSTN | Periostin |
| ROC | Receiver Operating Characteristic |
| mCRPC | metastatic Castration-Resistant Prostate Cancer |
| M±SD | Mean ± Standard Deviation |
| siPOSTN | POSTN-targeting siRNA |
| STAS | spreading through air space |
| TCGA | The Cancer Genome Atlas |
| TOP2A | Topoisomerase IIA |
| WGCNA | Weighted Gene Co-expression Network Analysis |

3. **Figure 1 has one area with red fonts on green background, which is difficult to read use white or yellow on all dark backgrounds.**

Response:

Thank you for your careful review. We have remade Figure 1 according to your suggestion: changing the red/green background color scheme in the original image to white/dark green background (with higher contrast), and uniformly checking all dark background areas to ensure that the text is white or yellow, meeting the accessibility reading standards.

Modified figure 1：

4. **Figure 2A fonts are too small on the legends.- increase size to easily readable/ figure 2D show the green negative correlations higher to match the red intensity.**

Response:

We have revised Figure 2 and regenerated the high-definition image according to the following two points to ensure compliance with the journal display standards:

Enlarge the legend font uniformly to ≥ 8 pt (the journal requires the minimum readable font size), and maintain consistent font thickness; Synchronize and adjust the legend spacing to avoid crowding.

Increase the saturation of the green color scale to the maximum intensity (100%) that is positively correlated with the red color, and use a symmetrical color scale (- R to+R) to ensure that the negatively correlated areas are visually equally prominent as the positively correlated areas.

Modified figure 2：

5. **Figure. 3B and Figure 4E show P values.**

Response:

Thank you for your attention to the details of the chart. We have annotated the P-values related to Figure 3B and Figure 4E.

Modified figure3 and figure4：
